# Commercialisation and entrepreneurship – an academic viewpoint

**DOI:** 10.1038/s41467-022-35568-9

**Published:** 2022-12-21

## Abstract

We spoke to Professor Kylie Vincent – professor of inorganic chemistry at the University of Oxford, co-founder of HydRegen Ltd, and Academic Champion for Women in Entrepreneurship – about turning academic research into industrial products.

**Figure:**
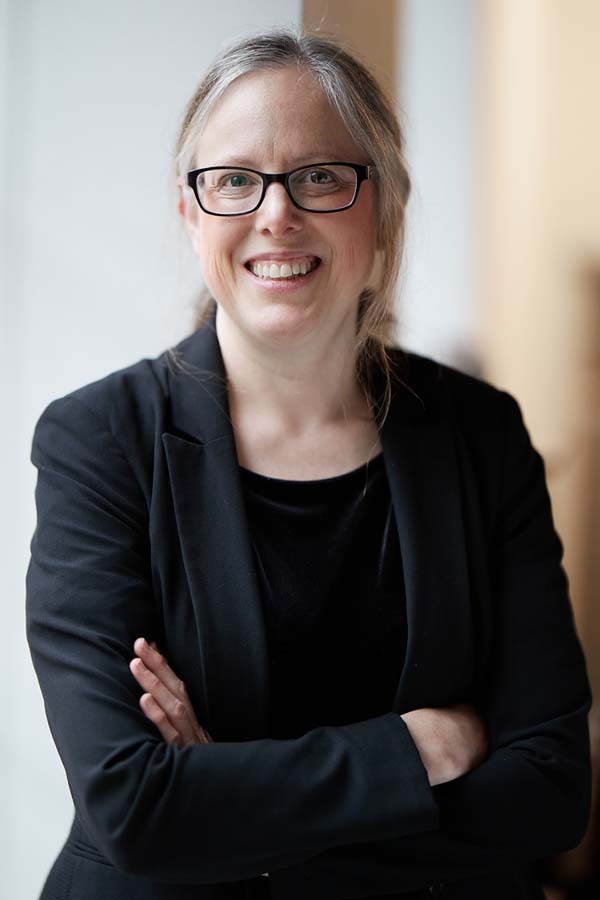
Prof Kylie Vincent Ian Wallman

In your view, what are the barriers to getting research from the lab to the market?

Firstly, there is often a long and costly research and development journey needed to get from lab concept to market. At the point where academic discovery is published, the demonstration of a new concept is typically small-scale, tested under a limited set of operating conditions and may use parts or chemicals that are uneconomical, unsafe or impractical to scale up: the innovation is still at a low technology readiness level. The funding stream for further translation towards market is not always clear because traditional academic research grants may not fund scale-up or prototyping, yet spinning out from an academic institution too early, with private investment, brings a greater risk of failure. There is increasing awareness of the need for translational funding, and the benefits that it can provide for facilitating successful commercialisation of academic discovery.

Secondly, innovation arising from academic research may yield an exciting new concept but it can take a long time to work out where this could best be applied. Take, for example, a new surface coating which might have hundreds of potential applications. Each field of application is likely to require improvement of different aspects such as robustness, longevity, thermal-stability or cost-effectiveness. There is a complex and circuitous market-discovery journey needed to work out the most promising application to target first, and this will need ongoing evaluation as research and development targets succeed or fail, and in response to shifts in the market or end-user demand.

Finally, for academics who are new to research commercialisation, the pathways to spinning out a company or licensing a technology can seem opaque and daunting. There is a new jargon to grapple with – ‘angels’, ‘venture capital’, ‘exit strategies’ and ‘elevator pitches’, for example. New ways of presenting the research innovation are required.

Is academia in general becoming more aware of, and involved in, the development and commercialisation of science?

The growth of technology transfer offices within academic institutions has greatly improved the support on offer to academics for commercialisation of scientific discovery. Graduate students are now frequently offered a wide array of training opportunities in entrepreneurship and intellectual property, and this is translating into a much more entrepreneurially-minded generation of researchers who will be better-placed to take discoveries towards market. There is still work to done in how commercialisation activities are recognised as part of academic workload.

The valley of death is a phrase often used to describe the funding gap for mid-level technologies: research that is not novel enough for basic science/discovery grants, but neither is it ready for commercialisation. Do you feel that this is a problem for translational research?

The so-called valley of death is still a real challenge for translational research. As I noted earlier, innovations arising from academic research are likely to require a long development pathway and extensive market research and end-user engagement before it is possible to pin-point a target market opportunity. This can represent a lengthy and expensive development phase which is likely to be ill-suited for traditional academic grant funding, and yet be seen as risky for private investment. Luckily, funding bodies are increasingly recognising the importance of funding proof-of-concept studies and impact-generation from academic research, so there are opportunities to carry out some of this development phase within the academic environment and hence lower the risks a little before spinning out into the commercial world.

What role can journals play in helping to bridge the valley of death?

The advent of funding schemes for proof-of-concept or impact-focussed academic projects means that more of the early stage translation of research innovation is being done within an academic environment where publication is an expected output. An openness by journals to publish studies that target scale-up, prototyping, user evaluation, for example, will support researchers seeking to de-risk a technology in the academic environment rather than trying to commercialise too early.

What are your thoughts on patenting versus publishing when developing new technologies?

It is a common misconception that there must be a choice between publishing or patenting. Graduate students are often worried that an application-focussed project will impact their publication record. In reality, academic research that is worthy of patent-protection is also likely to lead to highly-read publications. It is, however, critical to get the order right, so that promising innovations are patent-protected *before* disclosure via conference presentations, discussions or publications. Patent lawyers will often work to tight deadlines to draft patent claims if a publication is on the horizon.

As a champion for women in entrepreneurship, what do you think needs to be done to remove barriers for women in research commercialisation?

In my view, a critical step to make entrepreneurship more attractive to women is to ensure that entrepreneurial culture is inclusive, flexible and supportive. The off-putting stereotype of entrepreneurship as a round-the clock commitment still persists. Nevertheless, I see an inspiring generation of women carving out new models for how to balance executive level roles with life and family. Rather than expecting women to fit into out-dated ways to run companies, we need men *and* women to work together to fix the system. We can then celebrate what diversity in entrepreneurship can bring: more compassionate boards, different approaches to risk and recruitment, differing growth models, new attitudes to corporate responsibility.

Given that women are typically under-represented in both technology and management areas, taking a leading role in technology commercialisation can feel isolating for women. Peer mentoring can be valuable in connecting women with others in similar roles, as well as helping to alleviate the ‘imposter syndrome’ often experienced by women in roles where they are in an obvious minority. Enterprise training courses and board shadowing programmes targeted at women build skills and confidence, and also embed entrepreneurial women in a relevant network. Visible celebration of the women successfully commercialising research is important.

I don’t think we yet have a sufficient understanding of all the barriers to increasing diversity in research commercialisation. Continuous monitoring of diversity data at all stages in the pipeline of technology innovation remains really important. It is very clear that there are huge gains to be made by broadening the pool of innovators taking research concepts through to market, both in terms of the range of technologies commercialised, but also the new ways of thinking about entrepreneurship.

